# Evolutionary Origins of the Oligodendrocyte Cell Type and Adaptive Myelination

**DOI:** 10.3389/fnins.2021.757360

**Published:** 2021-12-01

**Authors:** Jacob H. Hines

**Affiliations:** Biology Department, Winona State University, Winona, MN, United States

**Keywords:** oligodendrocyte, neural progenitor, oligodendrocyte progenitor cell, evolution, gene regulatory network, myelin plasticity, adaptive myelination

## Abstract

Oligodendrocytes are multifunctional central nervous system (CNS) glia that are essential for neural function in gnathostomes. The evolutionary origins and specializations of the oligodendrocyte cell type are among the many remaining mysteries in glial biology and neuroscience. The role of oligodendrocytes as CNS myelinating glia is well established, but recent studies demonstrate that oligodendrocytes also participate in several myelin-independent aspects of CNS development, function, and maintenance. Furthermore, many recent studies have collectively advanced our understanding of myelin plasticity, and it is now clear that experience-dependent adaptations to myelination are an additional form of neural plasticity. These observations beg the questions of when and for which functions the ancestral oligodendrocyte cell type emerged, when primitive oligodendrocytes evolved new functionalities, and the genetic changes responsible for these evolutionary innovations. Here, I review recent findings and propose working models addressing the origins and evolution of the oligodendrocyte cell type and adaptive myelination. The core gene regulatory network (GRN) specifying the oligodendrocyte cell type is also reviewed as a means to probe the existence of oligodendrocytes in basal vertebrates and chordate invertebrates.

## Introduction

**FIGURE 1:**
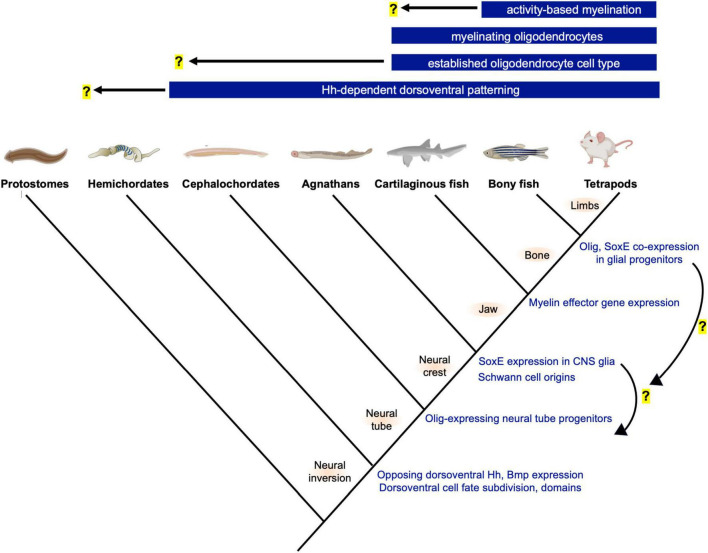
Phylogeny and innovations related to neural tube patterning and the evolution of oligodendrocytes. Top bars show the earliest known origins for novelties related to oligodendrogenesis and myelination. Hemichordates and cephalochordates such as acorn worms and amphioxus show similarities in neural tube dorsoventral patterning and progenitor domain subdivision. Future studies are needed, but the gradual changes to oligodendrocyte-associated gene expression (right, blue font) suggest step-wise GRN assembly and raise the possibility that the oligodendrocyte cell type preceded the evolution of the myelinating phenotype. Illustration generated using BioRender (www.biorender.com).

Vertebrate central nervous systems (CNSs) possess several distinct glial cell types that are essential for CNS functions. Oligodendrocytes are most frequently associated with myelination, but also perform other functions that are less well characterized. For example, oligodendrocyte lineage cells provide metabolic support to axons, influence axonal and dendritic growth, form synapses with neurons, regulate inflammation and angiogenesis, produce extracellular matrix to form perineuronal nets, and influence blood-brain barrier function (Mathis et al., 2003; Doretto et al., 2011; Saab et al., 2013; Yuen et al., 2014; Akay et al., 2021; Kohnke et al., 2021). The evolutionary origins of the oligodendrocyte cell type and functions of primitive oligodendrocytes are unknown, and no unifying or well-accepted model exists. Because oligodendrocytes are so closely associated with myelination, it may be assumed that oligodendrocytes evolved to myelinate axons and that the cell type did not precede that of myelination. However, increasing knowledge of non-myelinating functions such as the recent discovery that teleost oligodendrocyte progenitor cells (OPCs) regulate circuit development in the visual system raise the possibility that primitive oligodendrocytes may have initially served to perform functions other than widespread myelination of CNS axons (Xiao et al., 2021). This idea was originally proposed by Richardson et al. (1997). In comparison to the origins of the oligodendrocyte cell type, much more is known about the evolutionary origins of myelin. All gnathostomes (jawed vertebrates) possess myelin that displays similar structure and molecular composition. Based on available evidence, myelinating oligodendrocytes first emerged in an ancestral jawed fish prior to the divergence of cartilaginous and bony fish (Figure 1). The evolution of myelination is not the primary focus of this manuscript but has been reviewed elsewhere (Schweigreiter et al., 2006; Hartline, 2011; Zalc, 2016; Knowles, 2017; Rey et al., 2020).

If vertebrate myelin evolved in ancestral hinge-jawed fish, did oligodendrocytes evolve coincidentally, or did the cell type emerge prior to the evolution of the myelinating phenotype? To date, an oligodendrocyte-like cell has not been characterized in organisms that lack myelin, but the CNS glia of invertebrate chordates and jawless vertebrates are incompletely characterized. Rather than making assumptions based on the absence of evidence, it will be beneficial to further expand initial characterizations of glia and oligodendrocyte-associated gene expression in invertebrate chordates and agnathans to either support or rule out their presence. Unlike myelin, which is structurally unmistakable in electron micrographs, identifying primitive oligodendrocytes is less straightforward. These cells may have been multifunctional and performed functions that were not retained by the gnathostome oligodendrocytes. Alternatively, primitive oligodendrocyte functions could be similar to the non-myelinating functions of gnathostome OPCs and oligodendrocytes. In comparison to myelination, these non-myelinating functions are less well characterized and do not necessitate specialized structures that would be morphologically identified as easily as myelin sheaths. In addition, the upstream transcriptional regulators and effector genes mediating oligodendrocytes’ non-myelinating functions are less well understood. In evolutionary studies, the core regulatory factors expressed by a cell type may be a more accurate indicators of cell identity than morphology and function (Wagner, 2015; Arendt et al., 2016). Therefore, to investigate the origins of the oligodendrocyte cell type, it is important to obtain a clear understanding of the core gene regulatory network (GRN) defining the gnathostome oligodendrocyte cell type as well as the genetic modules for each separable non-myelinating function. This information will benefit and add focus to the search for the origins of the oligodendrocyte cell type in chordates and jawless fish.

Relatively few studies have investigated glia and potential oligodendrocyte homologous cell types in living chordates or agnathans such as amphioxus, tunicates, hagfish and lamprey. However, some important studies and discoveries have been made, which together raise the possibility that the oligodendrocyte cell type may have evolved prior to the myelinating phenotype. Ultrastructural characterization of the amphioxus CNS revealed the presence of cells termed axial glia (Lacalli and Kelly, 2002). These cells bear striking resemblance to gnathostome oligodendrocyte progenitors and non-myelinating oligodendrocytes, but their molecular signatures and functions remain unknown. Lamprey possess a CNS glial population that contacts and wrap axons but expresses molecular markers that more closely resemble mammalian astrocytes (Weil et al., 2018). Whether this lamprey CNS glial cell type represents a primitive astrocyte, oligodendrocyte, a distinct and unrelated glial cell type, or a multifunctional glial cell from which gnathostome glia were derived all remain open possibilities. Perhaps the most intriguing discovery related to the evolution of the oligodendrocyte cell type is the observation of a lamprey ventral spinal cord glial population expressing SoxE factors homologous to gnathostome Sox8, Sox9, and Sox10 (Yuan et al., 2018). SoxE expression is also detected in undefined CNS cell types of the developing hagfish and amphioxus spinal cords (Ota et al., 2007; Jandzik et al., 2015). Although the structure, function, and gene expression profile of chordate and agnathan CNS SoxE^+^ cell populations are incompletely characterized, due to the master regulatory roles of SoxE family transcription factors in gnathostome oligodendrocyte development, these important findings raise the possibility that chordates and agnathans possess an oligodendrocyte-like cell type that lacks the ability to myelinate axons. This elevates the spinal cord as a CNS region of interest in the search for ancestral oligodendrocytes.

The purpose of this review is to evaluate evidence and potential models for the evolution of the oligodendrocyte cell type as well as the adaptive myelination phenotype. Taking into consideration evidence from both aquatic and terrestrial vertebrates, a working definition for the oligodendrocyte cell type based on core GRNs is proposed. I also propose models for how co-option, module integration, and acquisition of novel genes can explain the evolution of the adaptive myelination phenotype. Finally, the importance of increasing diversity of research organisms used to study oligodendrocytes and myelin is discussed as an essential means to understand the evolutionary mechanisms responsible for this novel cell type and its myelinating and non-myelinating functions.

## Defining the Gnathostome Oligodendrocyte Cell Type

Cell types have classically been defined based on structural and functional properties, but the availability of genetic profiling techniques such as single-cell RNA sequencing provide new means to investigate and define cell identities. When cell morphology or function are used, it is important to recognize that cells are dynamic systems that may show a range of phenotypes depending on the context. For example, many cell types change cellular state over time or in response to environmental stimuli (Kin, 2015; Xia and Yanai, 2019). Due to this phenotypic plasticity, the challenges associated with defining a cell type based on structures and phenotypes are even more pronounced when evaluating homologous cell types between species. For instance, the notion that photoreceptors evolved multiple times was initially based on morphological evidence. This was later overturned by evidence of molecular homology across metazoans, instead supporting a common evolutionary origin (Arendt, 2008).

Oligodendrocytes provide another example of the issues and challenges associated with defining cell types based on morphologies and functions. The oligodendrocyte cell type was discovered by del Rio Hortega (1921) as a cell with few branches, which was based on observations of myelinating oligodendrocytes (Pérez-Cerdá et al., 2015). Oligodendrocytes have historically been defined by the singular function of myelination (Baumann and Pham-Dinh, 2001; Kettenmann and Ransom, 2013). Excluded from this definition are OPCs and pre-myelinating oligodendrocytes, which within this narrow definition, are immature cells destined to become oligodendrocytes. In further conflict are emerging discoveries that OPCs, pre-myelinating oligodendrocytes, and myelinating oligodendrocytes perform functions unrelated to myelination. It is important that a revised definition of the cell type includes all oligodendrocyte lineage cells irrespective of their developmental stage or cellular state. This is especially important in an evolutionary context because the morphology and functions performed by ancestral oligodendrocytes are unknown.

What are the defining properties of oligodendrocytes? This is challenging from a morphological standpoint because oligodendrocyte lineage cells may exhibit a simple bipolar morphology, may develop a complex and branchy morphology with dynamic process extensions, or may possess myelin sheaths (Pfeiffer et al., 1993; Baumann and Pham-Dinh, 2001; Kirby et al., 2006). Ultrastructurally, myelinating oligodendrocytes may be distinguished from other CNS glia by their smaller size, chromatin density, absence of intermediate filaments, and abundance of microtubules. However, whether these structural hallmarks are inclusive or defining of non-myelinating oligodendrocytes remains to be established. The heterogeneity of oligodendrocytes poses a challenge in defining oligodendrocytes based on morphological and functional properties. As stated by Dimou and Simons (2017), the phenotypic diversity and plasticity blurs the distinction between cell types and cell states. Defining a non-myelinating cell as an oligodendrocyte based on morphology alone is not straightforward or ideal, especially when comparing potentially homologous or analogous cell types between species.

Defining cell types based on molecular signatures offers an additional and complementary approach. In contrast to cellular structure and phenotypes, which change depending on the various states that cells encounter and can blur when comparing homologous cell types, the transcription factors expressed by a cell type tend to show strong evolutionary conservation (reviewed by Arendt et al., 2016). A subset of these factors form a core GRN, also referred to as a core regulatory complex (CoRC), that defines the cell type irrespective of changes to structure or function that may occur under various contexts or cellular states (Peter and Davidson, 2011; Arendt et al., 2016). Therefore, a core GRN refers to a set of transcription factors and accessory proteins that establish the gene expression profile for the cell-type. By definition, a core GRN is a stable complex that is maintained independent of cell states and can be stable over evolutionary timescales (Wagner, 2007; Peter and Davidson, 2011; Arendt et al., 2016; Xia and Yanai, 2019). The core GRN for a cell type may have autoregulatory loops, induce expression of terminal selectors that will drive effector or realizer gene expression responsible for phenotypes, and expression of genes that suppress other cell type core GRNs (Kin, 2015). A predominant current view in evolutionary cell and developmental biology is that the core GRN can be more stable than cell morphology over evolutionary time. While there are instances where this is the case (reviewed by Arendt et al., 2016), this has not been fully investigated for all cell types, and the emergence of more transcriptome data in future years will offer insight. This manuscript moves forward with the assumption that the oligodendrocyte core GRN is likely to be conserved, and therefore can be used as an identifier in the search for oligodendrocytes in basal vertebrates and invertebrate chordates. However, it is worth considering the alternative possibility that transcriptomes for ancestral oligodendrocytes may not be conserved, and that oligodendrocytes in different species could exhibit similar cell morphology and functions despite using different core GRNs.

Much effort has been dedicated to the understanding of the GRN for oligodendrocyte differentiation (reviewed by Emery and Lu, 2015; Li and Richardson, 2015; Cantone et al., 2019; Elbaz and Popko, 2019). The primary focus in these studies is on myelin gene expression as a readout of differentiation rather than the core GRN defining the oligodendrocyte cell type irrespective of the myelin differentiation program. The maturation into a myelinating oligodendrocyte represents one possible state for the oligodendrocyte cell type. However, because myelin gene expression is restricted to these states, components of the oligodendrocyte GRN specific to myelination should not be considered part of the core GRN. Rather, this type of specialized feature of a cell type is a secondary phenotypic module that operates in addition to the cell type core GRN, also referred to as an apomere (Arendt et al., 2016; Xia and Yanai, 2019). I therefore propose that myelination and its underlying GRN is a specialized phenotypic module, and that the transcriptional regulators and network responsible for myelination are distinct but not necessarily mutually exclusive from the core GRN defining the oligodendrocyte cell type. If open to the possibility that primitive oligodendrocytes lacked the myelinating phenotype, then it is important to separate the core GRN defining the cell type from the genetic module responsible for the myelinating potential. Failure to do so may impede the search for the evolutionary origins of oligodendrocytes.

If the transcriptional regulators that mediate myelination but not oligodendrogenesis are set aside, what is left to define the oligodendrocyte core GRN and cell identity? One obvious factor is Olig2, which is a primary mediator of oligodendrocyte production in zebrafish, chick, mouse, and human stem cells (Sun et al., 2001; Zhou et al., 2001; Lu et al., 2002; Park et al., 2002; Zhang et al., 2005). In the developing spinal cord, Olig2 is expressed by multipotent progenitor cells in the novel precursor to motor neurons (pMN) domain. Here, Olig2 expression represses expression of selectors such as Nkx2.2 and Irx3, which define neighboring progenitor cell domains and specify alternative cell fates (reviewed by Dessaud et al., 2008; Kessaris et al., 2008). Cell fates of Olig2 expressing spinal cord progenitors include motor neurons, interneurons, radial glia, oligodendrocytes, and motor exit point (MEP) glia (Park et al., 2004; Dessaud et al., 2008; Smith et al., 2014; Fontenas and Kucenas, 2021). In mice, loss of Olig2 results in a complete loss of spinal cord oligodendrocytes and a severely reduced population in the forebrain (Lu et al., 2002). Similarly, in zebrafish, loss of Olig2 results in the complete absence of spinal cord and hindbrain oligodendrocytes (Park et al., 2002; Zannino and Appel, 2009). Together, these studies indicate that Olig2 plays a key role in specifying oligodendrocyte cell type identity and should be included in the oligodendrocyte core GRN.

Several members of the Sox family of HMG-box transcription factors regulate oligodendrocyte development in ways that appear to be conserved across gnathostomes, making Sox transcription factors candidates for inclusion in the oligodendrocyte-defining core GRN. Members of the SoxE group including Sox8, Sox9, and Sox10 participate in both oligodendrocyte specification and myelination. Sox10 is widely recognized for its role in oligodendrocyte development and is expressed by OPCs and differentiating oligodendrocytes. Paradoxically, *sox10* mutants produce abundant oligodendrocyte progenitors in both mice and zebrafish but show deficits in oligodendrocyte differentiation and myelination (Stolt et al., 2002; Takada et al., 2010). These findings initially suggested Sox10 may not participate in early oligodendrocyte development, and instead, that Sox10 functions exclusively in differentiation toward myelination. However, subsequent single and double mutant analysis indicated important roles for Sox10 in generation of OPCs when *sox9* is also deleted, suggesting some functional redundancy in SoxE functions (Finzsch et al., 2008). Similarly, both Sox8 and Sox9 function in early oligodendrocyte development in mice and double mutant analysis indicates some overlap in functions (Stolt et al., 2005). Regarding conservation across gnathostomes, both Sox9 and Sox10 participate in oligodendrocyte development in zebrafish (Esain et al., 2010; Takada et al., 2010). Although we have limited insight into glial development in agnathans, SoxE factors may play similar roles in lamprey spinal cord development (Yuan et al., 2018). Due to their important roles in oligodendrogenesis, early OPC development, and established functions in both teleosts and mammals, SoxE factors should be considered as central components of the core GRN defining oligodendrocyte identity.

Ascl1 is a basic helix-loop-helix transcription factor that regulates oligodendrocyte production in mice (Parras et al., 2004, 2007; Vue et al., 2014). Zebrafish paralogs Ascl1a and Ascl1b are expressed in the developing neural tube, and Ascl1a mRNA expression has been identified in both neurons and oligodendrocyte lineage cells (Allende and Weinberg, 1994; Scott et al., 2021). Whether zebrafish Ascl1a functionally participates in oligodendrogenesis has yet to be determined. Nkx6.1 performs similar early roles in mouse oligodendrocyte development including the positive regulation of Olig2 expression (Liu et al., 2003). Whether this mode of regulation is common to all gnathostomes is unknown. Zebrafish Nkx6.1 is expressed in neural tube progenitors but its expression and function in the oligodendrocyte-lineage has not been examined (Guner and Karlstrom, 2007). Future studies in anamniotes investigating the roles for Ascl1 and Nkx6.1 in oligodendrocyte production will be important steps toward including or excluding these transcriptional regulators in the gnathostome oligodendrocyte core GRN.

Overall, more than two dozen transcription factors have been identified that regulate oligodendrocyte development in various ways. For example, Myrf, Nkx2.2, Olig1, Zfhx1b/Sip1/Zeb2, Nfatc2, Tcf7l2, Zfp488, Zfp24/Zfp191, and Sox17 promote differentiation, whereas Sox5, Sox6, Hes1, Hes5, Id2, and Id4 negatively regulate differentiation (reviewed by Emery and Lu, 2015; Elbaz and Popko, 2019). These factors appear to be more closely associated with the process of differentiation into myelinating oligodendrocytes rather than the specification or maintenance of OPCs, which distinguishes these factors from Olig2 and SoxE proteins, and potentially from Ascl1 and Nkx6.1. Therefore, while these differentiation and myelin-associated factors are certainly important regulators of oligodendrocyte development, they may be less relevant as identifiers of the oligodendrocyte CoRC identity in an evolutionary context because if ancestral oligodendrocytes were non-myelinating, these factors may not be expressed or be expected to perform essential functions in extant invertebrate chordates or jawless vertebrates.

Collectively, available evidence supports a model whereby Olig2 and SoxE factors comprise the core GRN of the oligodendrocyte cell type across gnathostomes, with the potential additions of Ascl1 and Nkx6.1 needing additional studies. Accordingly, any CNS cell type sharing Olig2 and SoxE expression but lacking neuronal markers should be evaluated as a putative oligodendrocyte. Olig2 and SoxE factors likely offer an incomplete picture of the oligodendrocyte core GRN or CoRC, and it will be important for future studies to expand this working definition. Further investigation into mammalian pro-oligodendrocyte factors such as Ascl1 and Nkx6.1 in additional model organisms would strengthen this working definition. It is likely that futures studies that disconnect and distinguish the oligodendrocyte identity from the myelinating phenotype will expand this working definition of the oligodendrocyte core GRN. One potential issue is that this proposed gnathostome oligodendrocyte core GRN overlaps with the gene expression profile of spinal cord MEP glia, which also express Olig2 and Sox10 (Smith et al., 2014; Fontenas and Kucenas, 2021). This raises an interesting and potential evolutionary relationship between spinal cord motor axon ensheathing glia and oligodendrocytes that has been speculated but not yet thoroughly investigated (Li and Richardson, 2008). Gnathostome MEP glia and oligodendrocytes appear to be distinct cell type based on their different migratory trajectories and PNS/CNS residence, but it is possible that these cells could represent distinct forms or states of a single cell type. MEP glia but not oligodendrocytes are known to express Foxd3 and Wif1 (Fontenas and Kucenas, 2018). This information may be used to investigate distinctions, functional overlap, and the potential for related evolutionary origins of these potential sister cell types. Importantly, by extending the current working definition of the oligodendrocyte core GRN, cells observed in different species may be defined as homologous or primitive oligodendrocytes if they possess the same core GRN, even if their morphology or functions show less similarity to gnathostome oligodendrocytes.

## Models for Oligodendrocyte Cell Type Evolution

Since the origins of multicellularity, a plethora of distinct cell types have evolved to give rise to the complexity and diversity of life on Earth. In instances such as the evolutionary origins of neurons, similar cell types observed in distantly related species likely share common ancestral origins, and are therefore considered homologous (Kristan, 2016). This is not always the case, and in some instances, apparent similarities between cell types do not share a common ancestral origin. Instead, these similarities arose by convergent evolution (also referred to as cell phenotypic convergence) and are considered analogous but not homologous (Wagner, 2007; Arendt et al., 2016). One example of analogous cell types are ensheathing glia, which have been observed in various invertebrate and vertebrate groups. Despite their similar propensities to contact and wrap axons, ensheathing glia observed in different phylogenetic groups do not appear to share a common ancestral origin or molecular signatures (reviewed by Hartline and Colman, 2007; Hartline, 2011). As one of several ensheathing glial cell types, oligodendrocyte origins most likely trace back to an ancestral invertebrate chordate or basal vertebrate animal.

If all living cells can be traced back to the same ancestral cell, how does a novel cell type first appear in an organism when it was lacking in the parental generation? Different cell types show remarkably diverse cellular phenotypes, but there are common underlying mechanisms by which new cell types evolve. In sexually reproducing organisms, cell types are not directly passaged from parent to offspring. Instead, the genetic instructions responsible for producing all cell types are transmitted to offspring, and developmental processes are then responsible for interpreting and implementing these instructions to produce the proper cell types at the proper times, positions, and in the proper abundance. For a new cell type to evolve, changes to these genetic instructions must occur that result in new combinations of gene products expressed by individual cells. A new cell type is born if this novel genetic signature establishes a core GRN that defines and maintains a distinctive cellular identity (Kin, 2015; Arendt et al., 2016; Oakley, 2017). In this section, the principles of duplication, divergence, and co-option are applied to evaluate three separate models for the evolutionary origins of the oligodendrocyte cell type.

### Novel Progenitor Zone Model

**FIGURE 2:**
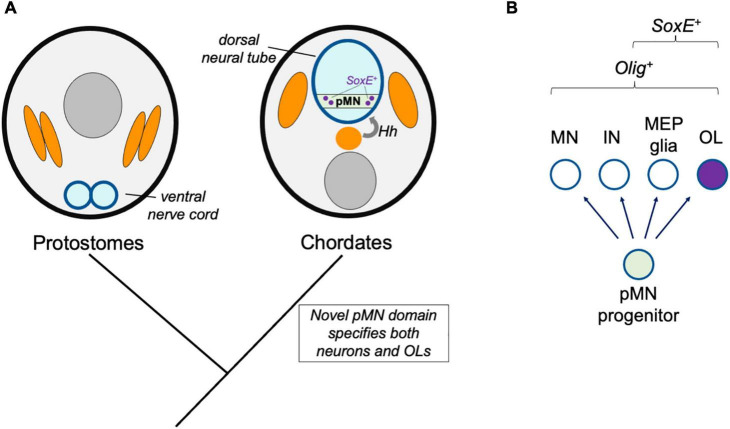
Novel progenitor zone model. **(A)** Hypothetical model for the origins of the oligodendrocyte cell type in ancestral chordates. Whereas protostomes possess a ventral nerve cord, the emergence of the dorsal neural tube and Hedgehog (Hh)-dependent patterning of neural tube progenitor zones corresponded to the emergence of the pMN domain (green) and its derivatives, including oligodendrocytes (OL). **(B)** This model predicts that all species with pMN progenitors produce a subset of derivatives that express oligodendrocyte core GRN factors including Olig and SoxE-family proteins.

The production of spinal cord oligodendrocytes by progenitor cells residing within the precursor of motor neuron (pMN) domain is a feature common of all gnathostomes observed to date. The pMN domain is one of several distinct progenitor cell populations that are patterned by opposing Hedgehog and BMP morphogen gradients along the neural tube dorsal-ventral axis (reviewed by Dessaud et al., 2008). In mice and zebrafish, oligodendrogenesis is blocked when the Olig-expressing pMN domain is experimentally disrupted (Lu et al., 2002; Park et al., 2002; Zhou and Anderson, 2002). Therefore, in species lacking what we would define as a pMN domain, such as protostomes, a homologous oligodendrocyte cell type may not be expected. The pMN domain and the dorsal neural tube are chordate novelties. The mechanisms of neurogenesis and nervous system centralization appear to be common to all bilaterians, but this is not the case for CNS patterning along the dorsal-ventral axis. Instead, dorsal-ventral patterning mechanisms appear to have independently evolved multiple times since the origins of bilaterians, and were more recently reinvented in ancestral chordates (Martín-Durán et al., 2018; Leung and Shimeld, 2019; Ren et al., 2020). In this “novel progenitor zone” model, the possibility that the new dorsal-ventral progenitor domains allowed for the emergence of the oligodendrocyte cell type is evaluated (Figure 2). A key prediction of this model is that once the Olig-expressing pMN progenitor domain evolved, this immediately caused the production of characteristic pMN cell type derivatives, including motor neurons and oligodendrocytes, and that all species with a pMN domain have oligodendrocyte-like glia.

The mechanisms patterning neural tube cell fates along the dorsal-ventral axis are well established and highly conserved between the mouse, chick, and zebrafish embryonic models. Notochord and floor plate-derived Hedgehog forms a morphogen gradient that establishes concentration-dependent expression of transcription factor profiles along the dorsal-ventral axis. Consequently, a population of ventral progenitor cells acquire Olig2 expression that marks the pMN domain (Dessaud et al., 2008). Implied by the name, these progenitors produce motor neurons as well as interneurons, oligodendrocytes, and MEP glia (Hall et al., 1996; Park et al., 2004; Fontenas and Kucenas, 2021). Graded Hedgehog signaling not only specifies ventral progenitor zones, but also influences the fates of more dorsally located progenitor zones in concert with an opposing BMP gradient (Dessaud et al., 2008). These morphogens induce a complex transcriptional network whereby a zone-defining transcription factor expression is maintained by positive feedback. The transcriptional network expressed within a zone also represses the transcriptional network associated with cell identities in other dorsal-ventral domains. In this way, progenitors exist in mutually exclusive states, and their daughter cells differentiate into a restricted set of cell types that associate with the parental progenitor cell transcriptional profile, zone of residence, and timing of differentiation. The complexity of this developmental patterning mechanism is thought to be responsible for the expansion of cell type diversity in vertebrate CNSs (Leung and Shimeld, 2019).

Expression of one of the core GRN factors for oligodendrocyte identity, Olig2, is dependent on Hedgehog signaling and the dorsal-ventral patterning GRN (Dessaud et al., 2008). Species lacking oligodendrocytes may not have the developmental niche producing Hedgehog adjacent to the neural tube or may lack critical components of the Hedgehog-responsive GRN. For example, protostomes lack a dorsal neural tube and notochord, and instead possess a ventral nerve cord that is patterned in a Hedgehog-independent manner. In contrast, hemichordates possess a dorsal nerve cord, and a short “collar cord” segment of the acorn worm dorsal nerve cord displays neural tube formation that resembles vertebrate neurulation (Miyamoto and Wada, 2013). This segment of the hemichordate neural tube is positioned next to Hedgehog-expressing cells, and the extent that Hedgehog signaling patterns the hemichordate neural tube remains to be determined. However, use of the Hedgehog-dependent patterning mechanism appears to be somewhat limited, because the ventral portion of the collar cord does not express transcription factors homologous to those expressed by vertebrate ventral progenitor zones. Additionally, a limited number of neuronal types form in a way that are not spatially organized across the dorsal-ventral axis (Miyamoto and Wada, 2013). Therefore, based on the predictions in this model, we would not expect to find a cell type with the oligodendrocyte core GRN in hemichordates.

One exception could be if dorsal neural tube progenitor cell populations are capable of producing oligodendrocytes. In amniotes, dorsal progenitor population specify about 15–20% of the total spinal cord oligodendrocyte population (Tripathi et al., 2011). These dorsal progenitors, marked by combinations of Dbx1, Pax7, and Msx3, are not Hedgehog-dependent or part of the pMN domain, but do acquire Olig2 and Sox10 expression at a later stage of development (Richardson et al., 2006). As is the case in the spinal cord, multiple sites of oligodendrogenesis have been identified in the amniote brain (Perez Villegas et al., 1999; Olivier et al., 2001; Richardson et al., 2006; Traiffort et al., 2016). One possibility is that this is an example of developmental homoplasy, where the same cell type can be generated by different progenitor cell populations. In this situation, different regulatory pathways are sufficient to achieve the same cell-type outcome (Graham, 2010). It has been proposed that the ventral pMN-derived oligodendrocytes are ancestral, and the dorsally derived oligodendrocytes are a later evolutionary specialization that may have helped to meet oligodendrocyte production and migratory demands when the vertebrate spinal cord and brain increased in size (Richardson et al., 2006). However, this is speculative, and it is also possible dorsal progenitor-derived OPCs are ancestral. In this case, that hedgehog-independent specification of OPCs in dorsal domains pre-dated the pMN-derived OPC population, which may have co-opted the GRN for dorsal-derived OPCs under the control of hedgehog signaling in ventral neural tube progenitors. In either scenario, these populations of oligodendrocytes of distinct progenitor cell origins appear to represent the same cell type, and can functionally compensate for one another (Kessaris et al., 2006; Tripathi et al., 2011). Although the ventrally derived OPC origins are most carefully examined in this manuscript, this possibility of dorsal neural tube origins and co-option by ventral progenitors should not be disregarded.

If hemichordates lack most features of vertebrate neural tube dorsal-ventral patterning, when did these mechanisms and the resulting progenitor cell zones evolve? In comparison to hemichordates, Hedgehog dependent neural tube patterning and progenitor domain division appears to be further evolved in the cephalochordate amphioxus. Similar to vertebrates, Hedgehog is expressed by notochord and floorplate cells, and its perturbation disrupts cell fates along the dorsal-ventral axis. Notably, amphioxus Hedgehog signaling most prominently impacts specification of a subset of Olig/Mnx-expressing motor neurons, which is also the case in vertebrates (Ren et al., 2020). This demonstrates that the rudimentary mechanisms inducing pMN-like progenitor zone fates is a chordate novelty that is not unique to vertebrates. Ventrally derived Hedgehog and a ventral Olig2^+^ cell population is also present in the agnathan lamprey spinal cord (Kano et al., 2010; Lara-Ramirez et al., 2019). Based on these observations, it is reasonable to speculate that the oligodendrocyte fate evolved as a consequence of this novel Olig-expressing, putative pMN-domain progenitor population. In this way, the evolution of new pMN-derived cell types would represent a new branch point on the neural tube cell division hierarchy, similar to the evolution of the neural crest as a novel progenitor population (Kin, 2015).

The periodic table of cell types model proposes that developmental lineage and fate maps can be used to predict species’ “missing” cell types and investigate their existence (Xia and Yanai, 2019). That amphioxus possess a vertebrate-like mode of neural tube patterning and putative Olig2^+^ putative pMN domain predicts that the pMN-derived cell fates of vertebrates may have evolved in an ancestral chordate prior to the divergence of cephalochordates. This possibility is under active investigation and there is currently no direct evidence to firmly support or reject this model. Consistent with this model is the observation that amphioxus possess a spinal cord glia population that resembles the morphology of gnathostome OPCs or pre-myelinating oligodendrocytes (Lacalli and Kelly, 2002). Because the Olig^+^ domain produces oligodendrocytes in gnathostomes, it would be interesting to determine if any amphioxus or lamprey CNS glia are derived from Olig-expressing progenitor cells. On the other hand, evidence that Olig-expressing progenitors exclusively generate neurons in these species would provide strong evidence refuting this “novel progenitor zone” model.

Although the pMN-derived oligodendrocyte cell type appears to be a chordate or vertebrate novelty, evidence suggests this is not the case for the motor neuron cell type, which is another pMN-derived cell type. In vertebrates and invertebrates with centralized nervous systems, motor neurons analogously extend axons CNS axons into the periphery to innervate and control musculature (Jung and Dasen, 2015). Differences in motor neuron specification accompanied or followed the neural tube inversion event, but noteworthy similarities also that suggest that vertebrate and invertebrate motor neurons are homologous cell types. One proposal is that chordates retained one subtype of non-chordate motor neuron that utilizes Hb9 (Thor and Thomas, 2002). Use of these ancestral transcription factors for motor neuron differentiation in chordates would have necessitated new upstream regulators in the new dorsal neural tube. Olig-family transcription factors are notable upstream regulators of motor neuron specification and pMN domain identity in chordates (Lu et al., 2002; Park et al., 2002; Zhou and Anderson, 2002). In contrast, the role of Olig factors in non-chordate neural development include roles in motor neuron axon targeting in *Drosophila* (Oyallon et al., 2012), as well as non-motor neuron expression and function in *Caenorhabditis elegans* CEPsh glia (Yoshimura et al., 2008; Felton and Johnson, 2014). Based on these findings, the downstream mechanisms of motor neuron differentiation appear to have ancient origins, but upstream mechanisms evolved to include hedgehog and Olig, which do not participate in motor neuron specification in non-chordates. In this first novel progenitor zone model, it is proposed that the novel use of hedgehog and Olig for induction of a pMN domain identity and motor neuron specification also produced oligodendroglia as an additional pMN-derived cell type.

How did the new chordate neural tube acquire the regulatory mechanisms specifying the diversity of CNS cell types seen in vertebrates? Consistent with the evolutionary theme of re-purposing old genes for new purposes, in chordate neural development, the ancient and multifunctional Hedgehog pathway was recruited to pattern novel cell fates along the CNS dorsal-ventral axis. Because Hedgehog participates in many developmental processes and GRNs, the new neural tube and patterning mechanisms likely co-opted pre-existing Hedgehog-induced pathways. In this new neural role, Nkx2, Nkx6, Olig, Irx, and Pax family transcription factors serve as Hedgehog-pathway target genes that specify and restrict cell fates. Furthermore, these transcription factors eventually evolved the ability to cross-repress one another, which was likely important in order to form mutually exclusive cell states and progenitor populations, to sharpen progenitor zone boundaries, and to expand the repertoire of CNS cell types. Evidence from hemichordates and cephalochordates suggest this new GRN and developmental patterning mechanism did not form at once, but instead underwent gradual or step-wise changes during chordate and vertebrate evolution (Ren et al., 2020). Although our current understanding of these events is very incomplete, this major chordate transformation in neural development likely involved a variety of mechanisms including co-option of pre-existing transcriptional modules, gene duplication, and evolution of new enhancers and transcription factor binding sites.

### Novel Precursor to Motor Neurons (pMN) Derivatives Model

**FIGURE 3:**
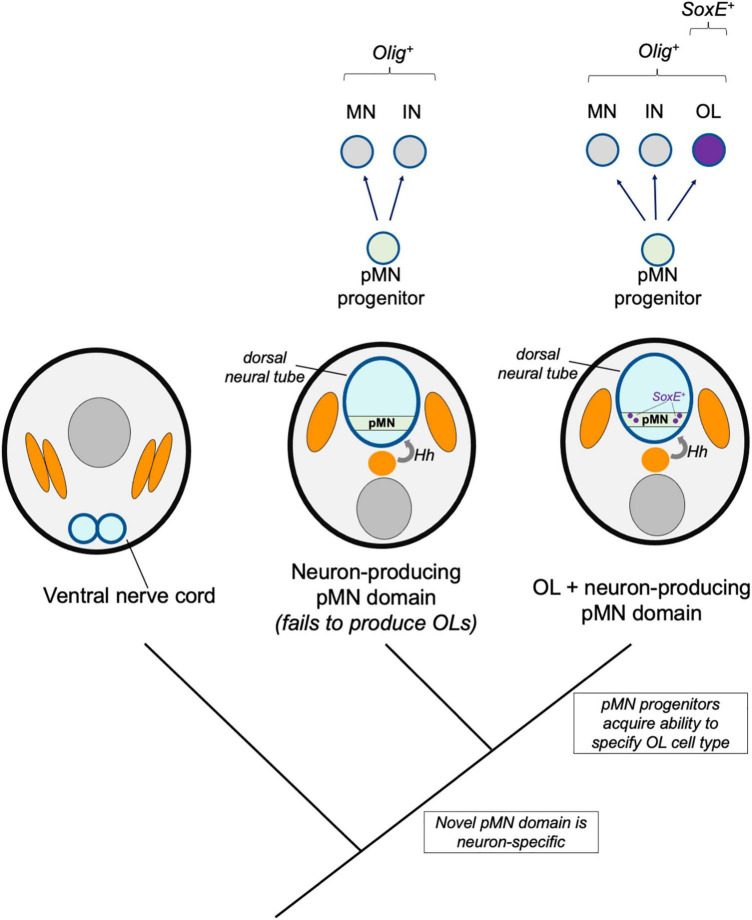
Novel pMN derivatives model. A hypothetical model shows stepwise changes to neural induction and patterning. A primitive Hedgehog (Hh)-dependent neural tube patterning mechanism induced an Olig-expressing pMN domain (green) which initially produced motor neurons (MN) and interneurons (IN). Subsequent recruitment of SoxE expression to a subset of Olig-expressing progenitors was responsible for the origins of the oligodendrocyte cell type (OL, purple).

The dorsal neural tube and its basic patterning mechanisms are hemichordate or chordate novelties, but cephalochordates and gnathostomes show differences in patterning mechanisms. Available evidence suggests gradual changes after the divergence of cephalochordate and vertebrate lineages (Ren et al., 2020). Although Olig^+^ pMN-like progenitor zones exist in cephalochordates (amphioxus) and agnathans (lamprey), it is unknown if these cells produce glia such as oligodendrocytes. In this model, we consider a scenario where the Olig-expressing pMN progenitors first arose as a neuron-producing domain, and that these progenitors subsequently evolved the ability to specify oligodendroglia (Figure 3).

It is possible and suggested by loss of function data in amphioxus that ancestors to all living chordates possessed a pMN domain and used Hedgehog and Olig in dorsal-ventral neural tube patterning prior to vertebrate evolution (Ren et al., 2020). However, this study revealed that the Hedgehog pathway plays a minor role in ventral neural tube development in amphioxus compared to its roles in zebrafish, chick and mouse embryos. Amphioxus Hedgehog regulates only a subset of the transcription factors defining dorsal-ventral progenitor domains in vertebrates and only influences the development of a subset of Olig and Mnx expressing motor neurons. In comparison, vertebrate Hedgehog regulates Pax and Irx family expression, and is required for Olig2 expression and development of all motor neurons types (Ericson et al., 1996; Dessaud et al., 2008). Together, these data suggest that the mechanisms specifying the ancestral chordate pMN domain and its motor neuron derivatives lack some mechanistic features observed in jawed vertebrates. Due to these major distinctions, it is reasonable to speculate that the ancestral pMN domain may have produced a set of cell types that did not include a primitive oligodendrocyte-like glia, and instead, that subsequent evolutionary changes to ventral neural tube patterning and pMN progenitor fates were responsible for the origins of the oligodendrocyte cell type.

The evolutionary transition from Olig^+^ pMN cells producing only neurons to specifying oligodendrocyte fates would likely be accompanied by the evolution of new Olig target genes. Based on the function of Olig in Drosophila motor neurons, Olig factors appear to have an ancient role in motor neuron development (Oyallon et al., 2012). This motor neuron role is also evident in amphioxus, but it is unclear whether the amphioxus Olig-expressing population also produces oligodendrocyte-like glia (Ren et al., 2020). If we accept that the oligodendrocyte core GRN includes Olig and SoxE factors, and that a cell must express both to be considered an oligodendrocyte, then the acquisition of SoxE expression in an Olig-expressing population is a major event in the history and genesis of the oligodendrocyte cell type.

The existence of an Olig and SoxE double-positive cell population in the amphioxus neural tube has not been carefully examined. Amphioxus OligA, OligB, and OligC are expressed by ventral progenitor population that appear to be homologous to the vertebrate pMN domain based on expression of motor neuron markers and a requirement for motor neuron specification (Ren et al., 2020; Bozzo et al., 2021). SoxE is expressed in the amphioxus neural tube (Jandzik et al., 2015), but whether it overlaps with Olig2 has not been carefully examined. It appears that the acquisition of ventral spinal cord SoxE expression occurred before the divergence of agnathans. This is likely because Olig and SoxE are expressed in the lamprey ventral spinal cord but overlapping expression in the ventral spinal cord has yet to be demonstrated in hemichordates or cephalochordates, which do have an Olig^+^ pMN domain (Ren et al., 2020). Therefore, it is plausible that the core GRN for oligodendrogenesis evolved by converting a pre-existing pMN domain into an Olig and SoxE double-positive progenitor pool during late chordate or early vertebrate development.

If primitive Olig-expressing pMN cells lacked SoxE expression, which evolutionary mechanisms could be responsible for novel SoxE expression in ventral, Olig-expressing CNS progenitors? In mammals, *soxE* (*sox10*) is a direct downstream target of Olig2. Induction of Sox10 expression is facilitated by an enhancer that is conserved between birds and mammals (Küspert et al., 2011). This same *sox10* enhancer, bound by Olig2 and driving oligodendrocyte expression in mice and humans, similarly induces reporter expression in zebrafish oligodendrocytes (Antonellis et al., 2008; Pol et al., 2013). This suggests that the Olig2 targeting of *sox10* also occurs in zebrafish, but orthologous enhancer sequences have not yet been identified. It would be of interest to explore deeper functional homology in amphioxus and lamprey.

SoxE expression in mammalian Olig^+^ cells is important for oligodendrogenesis, because deletion of two of the three *soxE*-family members caused a nearly complete loss of OPCs (Stolt et al., 2005; Finzsch et al., 2008). This suggests that the duplication of *soxE* produced paralogs with partially overlapping and redundant functions. As is the case in mice, loss of Olig2 in zebrafish blocks CNS Sox10 expression (Park et al., 2002), and loss of Sox10 alone perturbs differentiation but not OPC production (Takada et al., 2010). It would be informative to know if deletion of multiple zebrafish *soxE* paralogs blocks OPC production in a manner similar to that seen in mice, which would suggest that the Olig-SoxE axis is part of an ancestral core complex for gnathostome oligodendrogenesis. If this is not the case, it would instead indicate that recruitment of SoxE for early oligodendrocyte specification is a derived feature of some vertebrates and might not be expected in primitive oligodendrocytes. Because both Olig and SoxE expressing cell populations have been identified in cephalochordates and agnathans (Ota et al., 2007; Jandzik et al., 2015; Yuan et al., 2018; Lara-Ramirez et al., 2019; Ren et al., 2020), it will be exciting to learn whether these populations overlap, and to explore the cell fates, morphologies, and functions of these putative chordate glia.

In addition to Olig-dependent regulation of SoxE factors, in mammals, Olig1 and Olig2 regulate the expression many downstream genes involved in oligodendrocyte differentiation. It is likely that some novel Olig target genes other than *soxE* may be important for the unknown functions of primitive chordate glia. The evolution of new Olig target genes is also likely to be important for the evolution of the myelinating phenotype. For instance, in mammals, Olig2 regulates expression of transcription factors required for myelination (Nkx2.2, Nkx6.2, and Sip1/Zeb2) and effectors associated with myelination [Myelin basic protein (Mbp), Cldn11, Mog, Mag, and Mobp] (Yu et al., 2013; Darr et al., 2017). Investigation of Olig target genes, their functions, and change during chordate and early vertebrate evolution would be informative to better understand the origins and evolution of oligodendrocytes. According to this novel pMN derivatives model, we would expect to find basal chordate groups with an Olig-expressing pMN domain that produced neurons but not glia. Furthermore, we would expect a transition where the pMN domain of more recently diverged groups such as jawless or cartilaginous fish specified novel pMN-derived glia and evolved new Olig target genes.

### Sister Cell Type Model

**FIGURE 4:**
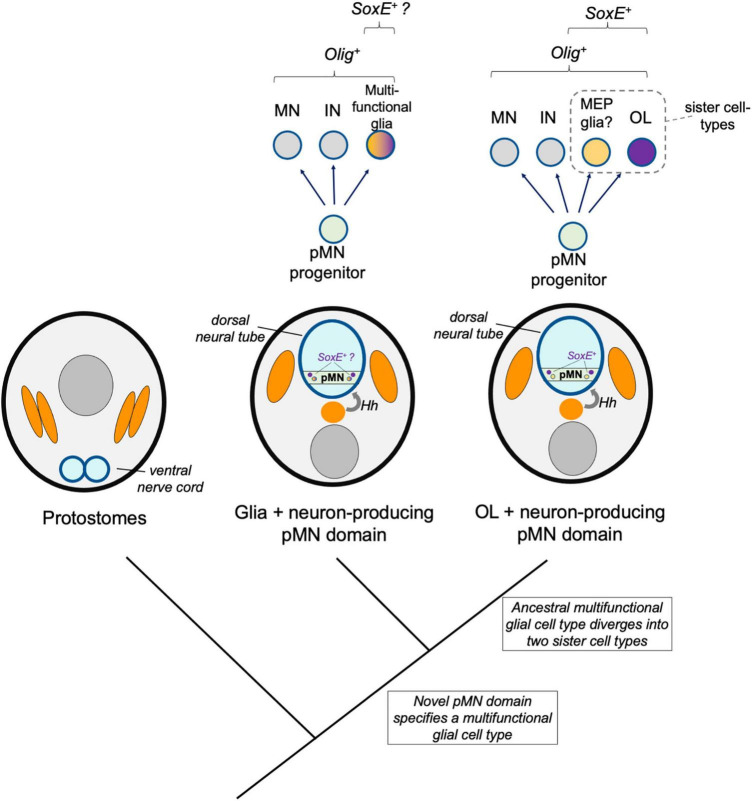
Sister cell type model. A hypothetical model shows a multi-functional pMN-derived glial cell type (orange–purple) that through duplication led to two novel cell types that diverged and specialized, including oligodendrocytes (OL, purple). A sister cell type to oligodendrocytes may be MEP glia, which also derive from Olig-expressing pMN progenitors, express SoxE factors, and myelinate motor axons in the periphery.

One mechanism thought to drive the evolution of novel cell types is the duplication of regulatory identities followed by divergence, specialization, and mutual repression of the alternative identity. In this way, multi-functional ancestral cell types diverge into two sister cell types. These sister cell types may lose features of the ancestral cells, may become more specialized, and may also co-opt new regulatory modules to take on new form and function. Here, an additional model is discussed whereby pMN-derived multifunctional glia existed prior to the origins of oligodendrocytes. Genetic changes occurred that drove formation of two distinct core regulatory networks, resulting in two sister cell types, one of which was the ancestral oligodendrocyte (Figure 4). A key distinction between the novel pMN derivatives and sister cell type models is the involvement of an intermediate multifunctional glia in the sister cell type model. In the sister cell type model, this multifunctional glia later diverged into at least two more specialized cell types, one of which was the oligodendrocyte.

The functions of the ancestral pMN-derived glial cell type cannot be addressed with current knowledge. This cell type may have been multifunctional, or it could more closely resemble the properties of a glial cell type found in extant vertebrates. If for example a multifunctional glia existed in an ancestral chordate prior to the divergence of the vertebrate lineage, we would expect it could be retained in living chordates such as amphioxus. This is possible and amphioxus possess glia including those with OPC-like morphologies (Lacalli and Kelly, 2002), but these chordate glia have uncharacterized functions and molecular signatures. If this multifunctional glial cell type produced two sister cell types through duplication (furcation) and divergence, we would expect a glial cell type would be retained in gnathostomes that performs some of the functions that this ancestral, multi-functional glia possessed.

One sister cell type candidate are motor glia. Due to the common origins and anatomical position of motor neurons and oligodendrocytes, Richardson et al. (1997) proposed that oligodendrocytes may have evolved from “motor glia” or to ensheath motor axons (Li and Richardson, 2015). This possibility is supported by evidence that zebrafish possess MEP glia that are generated by Olig2 and Nkx2.2 expressing progenitor population, exit the spinal cord, and myelinate motor axons on the peripheral face of the MEP (Smith et al., 2014; Fontenas and Kucenas, 2018, 2021). This raises the possibility that MEP glia and oligodendrocytes may be sister cell types that share a common ancestral Olig2^+^ multifunctional glial origin. This model is supported at the core GRN level, because the regulatory factors expressed by each cell type overlap. It is also proposed that sister cell types evolve ways to mutually repress the alternative fate. Although how this occurs is not yet clear, oligodendrocytes repress expression of Foxd3 and Wif1 (Fontenas and Kucenas, 2018), and it is likely that MEP glia repress transcription of oligodendrocyte-specific factors that have yet to be identified.

A second possibility is that rather than MEP glia, vertebrate astrocytes and oligodendrocytes are sister cell types. In this scenario, the ancestral multifunctional glia would have performed astrocytic and non-myelinating oligodendrocyte functions. At some point, the core GRN specifying this cell type diverged into primitive astrocytes and oligodendrocytes. This seems unlikely because we would expect to see some similarity in the core GRN for astrocytes and oligodendrocytes, but instead there is remarkably little similarity (Cahoy et al., 2008; Zhang et al., 2014). However, in support of this model, there is some commonality in the mechanisms specifying astrocytes and oligodendrocytes in teleosts. For example, zebrafish Sox9 influences production of both oligodendrocytes and astroglia in the developing hindbrain, and astrocytes homologous to mammalian astrocytes were recently characterized in zebrafish (Esain et al., 2010; Chen et al., 2020). It is possible but has not been investigated that chordate or basal vertebrate oligodendrocytes and astrocytes have more similar core GRNs, but these have diverged and specialized over the course of vertebrate evolution. Lastly, it cannot be excluded that the sister cell type to oligodendrocytes may have been lost over the course of evolution and that this cell population does not exist in extant species.

In order to support or reject the models described above it will be important for future studies to characterize CNS glia in invertebrate chordates and agnathans. These studies may reveal primitive oligodendrocytes or multi-functional glia in these species, or alternatively, may instead support the notion that the oligodendrocyte cell type evolved concurrently with the myelinating phenotype. Evidence that the pMN domain and oligodendrocyte-like CNS glia are present in in all chordates would most strongly support the “novel progenitor zone” or “novel pMN derivatives” models. Alternatively, evidence that neither oligodendrocyte-like cells or the myelinating phenotype existed in chordates or agnathans would support the idea that the cell type and myelination arose concurrently. This would be most consistent with the “novel pMN derivatives” or “sister cell type” models. A deep homology event is another possibility not considered in the models described above. For example, the oligodendrocyte cell type may have evolved through convergence to other pre-existing cell types, acquiring a small set of transcriptional regulators such as Olig2 and Sox10, rather than by co-opting a larger transcriptional network or through the divergence of sister cell types. Additional studies that investigate the genetic relationships between CNS glia across multiple chordate and basal vertebrate species will distinguish between these possibilities.

## Origins of Vertebrate Myelin and Adaptive Myelination

Oligodendrocytes are multifunctional cells that can exist in multiple cellular states. To transition to the myelinating state necessitates changes in gene expression resulting in the production and localization of gene products needed to form myelin sheaths. Myelin associated gene expression does not define the oligodendrocyte cell type identity or core GRN. Instead, the GRN for myelination is an accessory module specific to one of several cellular phenotypes exhibited by oligodendrocytes. Cell-type specific phenotypic modules such as myelination have also been referred to as apomeres (Arendt et al., 2016) and the purpose of this section is to evaluate potential mechanisms for the evolutionary origins of the apomeres for myelination.

Myelin was once thought of a fixed and unmodifiable insulation, but the discovery that myelination is adaptable to external experiences increases the molecular complexity and repertoire of gene products involved (reviewed by Monje, 2018; Foster et al., 2019; Bonetto et al., 2020; Pease-Raissi and Chan, 2021). Adaptive myelination (hereafter referred to as myelination) is a distinct cellular phenotype that is unique to oligodendrocytes in fish and mammals (Baraban et al., 2016). Myelination can be broken down into several components such as axon recognition and interactions, axon ensheathment, myelin wrapping and compaction, and activity-dependent structural and molecular remodeling. No other cell type deploys a phenotype using all of these components, but individually, these components can be found in other cell types. This is relevant because the evolutionary formation of apomeres can involve integration of multiple components. These components may be inherited from ancestral cell types or co-opted from other cell types. Therefore, to understand the evolutionary origins of myelination, it is important to consider which other cell types display similar phenotypes, and whether these cell types use similar genetic modules and effectors for the shared component or phenotype.

In addition to the obvious resemblance to other ensheathing cells, oligodendrocytes display functional and gene expression features that resemble the post-synaptic compartments of neuronal dendrites (Almeida and Lyons, 2013). Like neuronal synapses, mammalian oligodendrocytes can form both synaptic and non-synaptic contacts with axons (Bergles et al., 2000; Wake et al., 2015). Neuronal and neuron-oligodendrocyte contacts show molecular and physiological similarities including post-synaptic densities and post-synaptic currents (Bergles et al., 2000; Káradóttir et al., 2008; Maldonado and Angulo, 2015; Kula et al., 2019). The similarities between oligodendrocytes and dendrites also extend deeper, because in fish and mammals, both can undergo structural and molecular remodeling at sites of axon contact in response to changes in experiences and neural circuit activity (Wake et al., 2011; Makinodan et al., 2012; Hines et al., 2015; Baraban et al., 2018; Krasnow et al., 2018). Furthermore, like myelin sheath growth in fish and mammals, dendritic arbors and spines use the Akt-mTor pathway for cellular growth (Wood et al., 2013; Henry et al., 2017; Fedder-Semmes and Appel, 2021). Dendrites also enrich cholesterol and sphingolipid content in spines, which is similar to the distinctive lipid composition of myelinating membranes (Dotti et al., 2014). Together, these observations raise the possibility that myelinating oligodendrocytes are a hybrid of neuronal dendrites and ensheathing glia that have adapted and permuted ensheathing processes into multilamellar, compacted, and adaptable myelin sheaths.

**FIGURE 5:**
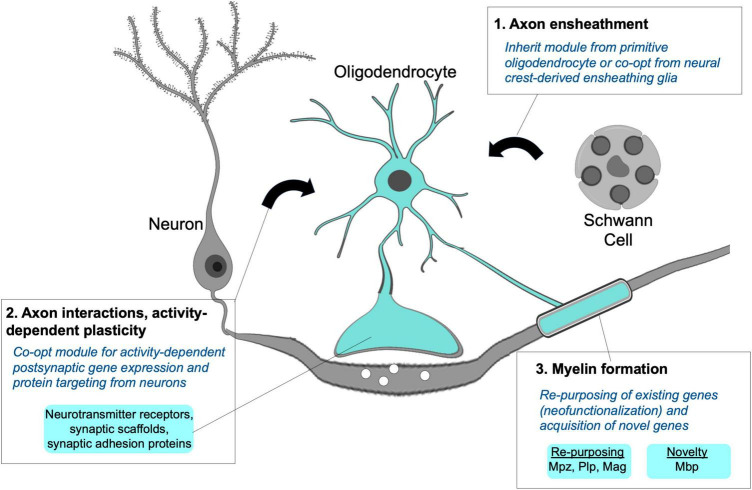
Module integration responsible for the origins of the myelinating phenotype. A hypothetical model proposes the myelinating apomere integrating modules for axon ensheathment (1), activity-dependent axon interactions and structural remodeling (2), and formation of compact myelin sheaths (3). Primitive oligodendrocytes may not have possessed all modular components, but their novel and combined use for myelination can be explained through co-option from neurons and non-myelinating Schwann cells along with the recruitment and re-purposing of existing genes required to form myelin sheaths.

Considering the similarities described above, it is logical to propose that the oligodendrocyte apomere for myelination evolved by integrating gene expression and protein targeting modules associated with ensheathing glia and neuronal dendrites in combination with the acquisition of novel myelin-associated genes that are unique to gnathostomes (Figure 5). Neuronal synapses have much earlier evolutionary origins than myelinating oligodendrocytes (Ryan and Grant, 2009). It is worth pointing out that the evolution of neuronal synapses involved integration of apomeres for both axon (pre-synaptic) and dendrites (post-synaptic) (Arendt et al., 2016), and both are relevant for axon-oligodendrocyte interactions. Because the synaptic apomeres pre-date the origins of myelinating oligodendrocytes, co-option of a pre-existing neuronal transcriptional module for expressing genes traditionally involved in dendrite function is a simple formula. For example, acquiring gene expression and protein composition typically associated with neuronal post-synaptic compartments could have enabled oligodendrocyte processes to recognize and adhere to axon-derived cues and adapt cell behaviors in response to changes in neuronal activity. This co-option and module integration within oligodendrocytes cannot completely account for the evolution of myelination, because neurons must also possess a compatible apomere for myelination to occur. However, glial co-option of post-synaptic mechanisms is a simple explanation because the neuronal (axonal) module was already in place. Furthermore, this neuronal (axonal) module needn’t undergo significant changes to interact with the same “dendritic” molecules that were co-opted for expression and targeting to oligodendrocyte processes.

Dendritic and myelinating processes are both highly specialized and comprise sets of proteins that are not expressed by most cell types in the vertebrate body plan. Despite this, the overlap in proteins typically designated as synaptic proteins that are present in both oligodendrocyte and dendritic processes is extensive. These have been nicely reviewed by Almeida and Lyons (2013) and more recently discussed by Hughes and Appel (2019). Multiple studies in fish and mammals demonstrate that axon-oligodendrocyte signaling through synaptic vesicle release and classic chemical neurotransmission-based mechanisms is functionally important for myelination (Wake et al., 2011; Hines et al., 2015; Mensch et al., 2015; Mitew et al., 2018). Both dendrites and gnathostome oligodendrocyte processes and sheaths possess and use similar synaptic scaffold and adhesion proteins. While their functions in glia are incompletely understood, these scaffold and adhesion proteins are required for myelin sheath growth (Almeida and Lyons, 2013; Hughes and Appel, 2019). Like dendrites, fish and mammalian oligodendrocytes express neurotransmitter and neurotrophin receptors, some of which have been linked to activity-dependent effects on myelination and myelin plasticity (Lundgaard et al., 2013; Yin and Hu, 2014; Geraghty et al., 2019). In zebrafish, oligodendrocyte responses to axonal cues appear to involve intracellular Ca^2+^ signaling (Baraban et al., 2018; Krasnow et al., 2018), which is similar to the well-established role of Ca^2+^ signaling in activity-dependent dendritic spine remodeling. Taken together, there are many similarities when examining how dendritic and oligodendrocyte process extensions respond to axon derived cues.

The transcriptional regulation of neuronal synapse-associated genes in oligodendrocytes is not well understood, but is likely to hold the key to understanding how oligodendrocytes evolved the ability to recognize axons and adapt their behavior to changes in circuit activity. For example, mammalian oligodendrocytes express factors with known roles in synaptic gene expression in neurons such as CREB and Nfat-family factors (Sato-Bigbee et al., 1999; Weider et al., 2018). This suggests that usage of “neuronal” cAMP and Ca^2+^-dependent transcription factors by oligodendrocytes could orchestrate synaptic gene expression in oligodendrocytes (Sato-Bigbee et al., 1994; Weider et al., 2018). An alternative possibility is that synaptic effector genes became target genes of transcription factors that were already expressed by oligodendrocytes. For example, enhancers of synaptic scaffolding, adhesion, and neurotransmitter receptor genes may have evolved binding sites for oligodendrocyte-specific transcription factors. In this way, the oligodendrocyte and neuronal regulation of synaptic genes would have evolved independently as an example of phenotypic convergence. Although possible, this scenario is less likely because it would involve coincident evolution of numerous enhancers. Instead, co-opted use of neuronal transcription factors to induce expression of synaptic genes in oligodendrocytes offers a simple formula for a new cell type to engage with existing axonal domains and undergo activity-dependent remodeling by adopting a pre-existing module.

As previously described, this co-opted neuronal module of the myelinating apomere may account for several mechanistic features of myelination, but alone, this module is insufficient to drive myelin sheath formation. Further integration with other gene products mediating axon ensheathment and multilamellar, compact myelin sheath formation was necessary for the evolution of myelination. It is unknown if ancestral chordate or agnathan CNS glia preceding the origins of myelination possessed ensheathing capabilities. Recent ultrastructural studies examining lamprey and amphioxus CNS glia identified axon-contacting glial processes, and it is possible these represent ancestral ensheathing glia from which the oligodendrocyte ensheathment module was derived or co-opted (Weil et al., 2018; Bozzo et al., 2021). Alternatively, lamprey possess non-myelinating Schwann cells that wrap peripheral axons (Fraher and Cheong, 1995). Therefore, co-option of an ancestral Schwann cell ensheathing module by CNS glia is possible, and if followed by concerted evolution, may explain the similarities in gene expression between Schwann cells and oligodendrocytes.

An additional and essential element of the myelinating apomere is the recruitment genes involved in forming compacted myelin sheaths. Re-purposing of existing genes through co-option cannot fully account for the myelinating phenotype because some genes necessary for myelination are not present in invertebrate chordate or jawless vertebrate genomes. A classic and well described example is Mbp, which is necessary for myelin formation and maintenance and is one of the most abundant proteins in myelin. The appearance of the *mbp* gene correlates with the emergence of myelination in gnathostomes (reviewed by Nave and Werner, 2021). Although Mbp function has not yet been tested in anamniotes, its acquisition was likely an early and requisite step for the evolution of myelin. The major effector proteins for myelination continue to be explored, and although *mbp* is an example of a novel gene, it is not surprising from an evo-devo perspective that most myelin effectors are not novel genes, and that gene re-purposing or neofunctionalization played a major role in the evolution of vertebrate myelination (Nave and Werner, 2021). This process involved a combination of changes including novel use of transcription factors as well as downstream effector genes, and evolution of the myelin gene regulatory program has been nicely reviewed elsewhere (Li and Richardson, 2015).

Notably, the apomere integration model proposed above assumes that vertebrate myelination has always been adaptive, and that there is no such thing as non-adaptive myelination. Substantial evidence for activity-dependent myelination in both mammals and teleost fish suggests that myelination was adaptive at least 380 million years ago in ancestors of modern bony fish. Because adaptive myelination has never been investigated in cartilaginous fish, it cannot be ruled out that the adaptive feature of myelination is an innovation that emerged during the relatively short evolutionary period between the divergence of cartilaginous and bony fish lineages. Future studies investigating adaptive myelination in cartilaginous fish such as sharks or skates would provide further insight into this question.

## Future Perspectives

The origins and evolution of the oligodendrocyte cell type and myelinating phenotype are among many unsolved mysteries in neuroscience. Few studies have directly addressed the origins of the oligodendrocyte cell type, but myelin evolution has been studied for several decades. Many discoveries have been made using rodent models along with some studies of myelin ultrastructure and biochemistry in lamprey, hagfish, shark, and ray CNSs (Kemali et al., 1983; Bullock et al., 1984; Waehneldt et al., 1984). More recently, the zebrafish model has become a valuable tool for the study of oligodendrocyte development and CNS myelination. As a representative species of bony fish, zebrafish studies have indirectly offered evolutionary insights. However, it is essential that additional research organisms be used to address these evolutionary questions. Without this, it will not be possible to understand the ancestral state and steps in the molecular evolution, and there are inherent risks in over-interpreting results from single species that may not accurately represent their branch on the tree of life. Given that living chordates represent hundreds of millions of years of evolution we should expect to find instances of derived traits, modules, and mechanisms. This can only be overcome by expanding the diversity of research organisms used.

One important area for future investigation is to determine which extant species have oligodendroglia, and which lack a homologous cell type. In addition to investigating expression of core regulatory factors described above, single-cell RNA-Seq profiling and comparisons between species are likely to be informative, but complex. Currently, it can be challenging to identify and classify distinct cell types within a single species. Investigations comparing species separated by tens or hundreds of millions of years of independent evolution will be difficult but are important endeavors. Important foundational work has been performed using amphioxus and lamprey. Both species offer expanding genomic resources, animal breeding, embryology methods, and tools for gene manipulation and reporter expression. For instance, seminal studies have identified CNS Olig and SoxE populations in amphioxus and lamprey that are yet uncharacterized (Yuan et al., 2018; Lara-Ramirez et al., 2019; Ren et al., 2020; Bozzo et al., 2021). It will be important to further investigate the identities, gene expression profile, and phenotypes exhibited by these cell populations, which could represent ancestral or sister cell types, or could be sources for co-option.

A second important area for investigation is to more fully characterize how the earliest gnathostome oligodendrocytes were specified and operated using comparative studies in extant species. It is not possible to understand how these cells arose without establishing the genes and core network that are involved and required. Fossil evidence has been used to propose that myelinating oligodendrocytes evolved prior to the divergence of placoderms, which of course cannot be confirmed or used to investigate the molecular evolution in living organisms (Zalc et al., 2008). Based on available evidence and among extant species, cartilaginous fish are the most basal vertebrate group that can be used to address questions related to the successful formula for myelinating oligodendrocytes. Myelination in little skates was recently characterized, and this model offers advantages that are frequently exploited for the study of neural crest and head skeleton evolution (Möbius et al., 2021). There are many questions that if addressed in cartilaginous fish would advance our understanding of myelin evolution. For example, which of the ∼30 transcription factors with established roles in mammalian oligodendrocytes are expressed by and functional in cartilaginous fish? Do cartilaginous fish use neuronal activity and adapt myelination to changes in experiences? Are synaptic adhesion and signaling molecules part of the core myelination program, or are these specialized features that evolved later in bony fish? How did the expression of myelination-associated transcription factors and key effector proteins change between the divergence of invertebrate chordates, agnathans, and cartilaginous fish, and how can enhancers or transcription factor binding explain these novelties? While some of these questions are being addressed in zebrafish, as teleosts, zebrafish are not basal vertebrates. Therefore, it is important to further develop investigation in cartilaginous fish to gain insight into the successful genetic formula for CNS myelinating cells in the most basal living vertebrates with CNS myelin.

It is unlikely that a cartilaginous fish model system will emerge that comes close to the high throughput and rapid generational cycles of bony fish models such as zebrafish. Therefore, much of our understanding of the successful formula for oligodendrocyte and myelin evolution will need to partially rely on bony fish. Zebrafish are widely recognized as a powerful model organism, but like any species, present some drawbacks. For instance, zebrafish and other teleosts are small and rapidly growing animals and also have a duplicated genome. Use of additional bony fish models as well as other non-mammalian vertebrates such as Xenopus is important to overcome these drawbacks and avoid over-reliance on a single model (Kaya et al., 2012). Rather than debating which model system is best or suppressing work on some to promote the emergence of others, members of the oligodendrocyte and myelin communities should unanimously welcome and value diversity in model systems used to study oligodendrocyte and myelin biology. First, this will offer greater insights into the origins and molecular evolution of oligodendrocytes. Second, we are frequently reminded in biological research that basic studies can provide powerful biomedical insights in ways that were previously unrealized. As was the case with CRISPR/Cas9 and many other basic investigations, the study of oligodendrocytes and myelin in non-model organisms is likely to provide more insight into human development and disease than may be anticipated.

## Author Contributions

The author confirms being the sole contributor of this work and has approved it for publication.

## Conflict of Interest

The author declares that the research was conducted in the absence of any commercial or financial relationships that could be construed as a potential conflict of interest.

## Publisher’s Note

All claims expressed in this article are solely those of the authors and do not necessarily represent those of their affiliated organizations, or those of the publisher, the editors and the reviewers. Any product that may be evaluated in this article, or claim that may be made by its manufacturer, is not guaranteed or endorsed by the publisher.
